# From Phenotypes to Spectrum: Rethinking RRMS, SPMS and PPMS in the Era of PIRA—A Framework Integrating PIRA, Smouldering-Associated Worsening, and Neurologic Reserve to Facilitate Earlier Recognition of Progression

**DOI:** 10.3390/neurolint18050086

**Published:** 2026-05-02

**Authors:** Georgi V. Vasilev, Sonya Ivanova, Ivan Milanov

**Affiliations:** 1 Clinic for Movement Disorders, University Multiprofile Hospital for Treatment in Neurology and Psychiatry “St. Naum”, 1113 Sofia, Bulgaria; s.ivanova@svnaum.com (S.I.);; 2 Department of Neurology, Medical University, 1431 Sofia, Bulgaria; 3 Clinic of Neurology, Multiprofile Hospital for Active Treatment “Uni Hospital”, 4500 Panagyurishte, Bulgaria

**Keywords:** multiple sclerosis, progression independent of relapse activity (PIRA), disability progression, neurologic reserve, biological ageing, smouldering disease activity, neuroaxonal injury, disease continuum, phenotype reclassification

## Abstract

The conventional classification of multiple sclerosis (MS) into relapsing–remitting, secondary progressive, and primary progressive phenotypes has long guided diagnosis, prognosis, and therapeutic decision-making. However, accumulating evidence indicates that disability accumulation frequently occurs independently of clinical relapses, challenging relapse-centric and phenotype-based models of disease evolution. The concept of progression independent of relapse activity (PIRA) has emerged as a clinically relevant framework capturing this phenomenon across MS phenotypes. In this state-of-the-art narrative review, we propose a spectrum-based reinterpretation of MS, integrating PIRA with concepts of smouldering-associated worsening and neurologic reserve. We highlight the heterogeneity of relapse-independent worsening, distinguishing transient from persistent PIRA, and discuss how ageing-related decline in compensatory capacity contributes to the clinical unmasking of progression over time. Within this framework, secondary progressive MS is redefined as the clinically recognizable accumulation of persistent relapse-independent worsening, while primary progressive MS is conceptualized as early predominance of clinically manifest progression due to limited reserve rather than a distinct disease entity. Finally, we examine diagnostic and therapeutic implications of a spectrum-based model in the contemporary era, emphasizing the limitations of relapse-centric treatment strategies and unmet needs in addressing progression-related biology. By reframing MS as a dynamic continuum shaped by the interaction between ongoing pathology and evolving neurologic reserve, this review aims to support earlier recognition of clinically meaningful progression and to inform more biology-aware approaches to disease monitoring and therapy.

## 1. Introduction

### 1.1. Classical Phenotypes and Their Clinical Utility

Since its formal clinical characterization, multiple sclerosis (MS) has been conceptualized and classified according to distinct disease phenotypes, most prominently relapsing–remitting MS (RRMS), secondary progressive MS (SPMS), and primary progressive MS (PPMS) [1].This phenotypic framework emerged from longitudinal clinical observations and represented a pragmatic attempt to describe heterogeneous disease courses in a reproducible and clinically meaningful way. The distinction between relapsing and progressive forms of MS provided an essential structure for diagnosis, prognostication, and therapeutic decision-making for decades [2,3,4].

From a clinical standpoint, phenotype-based classification has offered several important advantages. First, it facilitated a common language among clinicians and researchers, allowing standardized descriptions of disease course across centers and countries. Second, phenotypes became the backbone of clinical trial design and regulatory approval, with most disease-modifying therapies (DMTs) initially developed and tested specifically in RRMS populations. Third, large observational cohorts and registries adopted phenotype-based categorization as a core variable, enabling epidemiological comparisons and long-term outcome analyses [2,5,6,7].

Historically, the distinction between RRMS and progressive disease courses was grounded in readily observable clinical patterns: episodic neurological deterioration with partial or complete recovery in RRMS, versus steady accumulation of disability in SPMS and PPMS [8]. This dichotomy aligned well with early pathophysiological models emphasizing focal inflammatory activity as the dominant driver of clinical relapses, while progressive forms were considered biologically distinct, less inflammatory, and largely neurodegenerative in nature. Within this conceptual model, SPMS was viewed as a temporal evolution of RRMS, whereas PPMS was conceptualized as a separate entity from disease onset [9].

Despite their limitations, classical phenotypes remain deeply embedded in routine clinical practice. They continue to guide treatment eligibility, reimbursement policies, and therapeutic algorithms in many healthcare systems. Importantly, they also retain value as descriptive categories that reflect real and clinically relevant differences in disease expression at the population level. Any attempt to move beyond phenotype-based thinking must therefore acknowledge their historical importance and ongoing practical utility [2,7,10].

To clarify the distinction between formal MS diagnosis and subsequent clinical phenotyping, Table 1. summarizes the practical basis for classifying RRMS, SPMS, and PPMS in the era of the 2024 McDonald criteria.

### 1.2. The Problem: Delayed Recognition of Progression

While phenotypic classification has proven useful, it has become increasingly apparent that it is insufficient to capture the full complexity and temporal dynamics of MS. One of the most critical shortcomings of the traditional framework is the delayed recognition of disease progression, particularly the transition from RRMS to SPMS. In clinical practice, SPMS is almost invariably diagnosed retrospectively, often several years after irreversible disability accumulation has already occurred [1,11,12].

This diagnostic delay is partly driven by the tools traditionally used to assess disease worsening. The Expanded Disability Status Scale (EDSS), despite its widespread use, is heavily weighted toward ambulation and lower limb function, while being relatively insensitive to changes in upper limb function, cognition, fatigue, and other domains that substantially affect patients’ daily lives. Moreover, EDSS progression requires sustained worsening over time, further contributing to delayed recognition of subtle but clinically meaningful decline [13,14].

Equally important is the longstanding dominance of a relapse-centric disease model, in which inflammatory relapses are implicitly assumed to be the primary driver of disability accumulation. Within this paradigm, the absence of clinical relapses is often equated with disease stability, particularly in RRMS [15]. However, growing evidence indicates that patients may accumulate disability in the absence of overt relapses or new focal inflammatory activity detectable by conventional MRI. This disconnect between clinical relapses and long-term disability has challenged the traditional view that progression is confined to later disease stages or exclusively to progressive phenotypes [16,17].

The recognition of progression independent of relapse activity (PIRA) has fundamentally altered this perspective. PIRA highlights that disability accumulation can occur silently, without clear clinical relapses, and has been documented in a subset of patients from early stages of RRMS [16,17,18]. Importantly, this suggests that the biological processes driving progression precede the formal clinical diagnosis of SPMS, raising the possibility that progressive pathology is not a late complication, but rather an integral component of MS biology across phenotypes [19,20].

### 1.3. Aim and Scope of the Review

The aim of this state-of-the-art narrative review is to re-examine the classical phenotypic classification of MS in light of emerging evidence on PIRA and related concepts of smouldering disease activity and neurologic reserve. Rather than proposing the abandonment of RRMS, SPMS, and PPMS as clinical descriptors, this review seeks to position them within a dynamic spectrum model, in which phenotypes represent time-dependent expressions of shared underlying disease mechanisms.

Specifically, we aim to:

1. Summarize the Limitations of Traditional Phenotype-Based Classification in Capturing Early and Relapse-Independent Progression;
2. Examine PIRA as A Clinical Operationalization of Progressive Disease Biology Across MS Phenotypes;
3. Integrate PIRA with Biological Concepts Such as Smouldering-Associated Worsening and Neurologic Reserve;
4. Discuss the Diagnostic and Therapeutic Implications of Adopting A Spectrum-Based Framework for MS.

By reframing MS phenotypes through the lens of PIRA, this review seeks to provide a conceptual foundation for earlier recognition of progression and for more biologically informed therapeutic strategies, thereby bridging clinical classification with contemporary insights into MS pathophysiology.

## 2. Why Phenotypes Became Insufficient

### 2.1. EDSS-Centric Bias and Its Consequences

The Expanded Disability Status Scale (EDSS) has played a central role in the clinical assessment of multiple sclerosis for decades and remains deeply embedded in both routine practice and clinical research. Its strengths are well recognized: EDSS provides a standardized, longitudinally comparable measure of disability and captures ambulatory impairment with high granularity, which is particularly relevant in later disease stages. These features have made EDSS indispensable for natural history studies, clinical trials, and regulatory decision-making [4].

However, the dominance of EDSS has also introduced systematic bias in how disease progression is conceptualized and detected. By design, EDSS is heavily weighted toward lower limb function and walking ability, while being relatively insensitive to changes in upper limb dexterity, cognition, fatigue, mood, and other non-ambulatory domains [5,6]. As a result, clinically meaningful deterioration in these domains may occur without meeting conventional EDSS thresholds for confirmed disability worsening [5,6,7].

This imbalance has important implications for the recognition of progression. Early disability accumulation in multiple sclerosis often manifests as subtle declines in manual function, cognitive processing speed, endurance, or complex task performance—domains that are poorly reflected by EDSS, particularly in patients with preserved ambulation. Consequently, patients may be classified as clinically stable for prolonged periods despite ongoing functional decline, reinforcing the illusion of disease quiescence in the absence of relapses [5,8].

The EDSS-centric framework has also shaped the interpretation of treatment response. Therapeutic success has traditionally been equated with relapse suppression and stabilization of EDSS scores, implicitly assuming that absence of EDSS progression reflects control of the underlying disease process [9]. Increasing evidence challenges this assumption, demonstrating that disability accumulation may proceed independently of both relapses and overt EDSS change, particularly in early and mid-stage RRMS. Within this context, EDSS stability does not necessarily imply biological stability [2,10,11,12].

Importantly, the limitations of EDSS do not render it obsolete; rather, they highlight the risks of relying on a single outcome measure to define progression. An EDSS-centric view may delay recognition of progressive disease biology, bias phenotype assignment, and obscure early signals of progression that are clinically relevant but insufficiently captured by traditional scales [13,14]. This realization has been a major driver behind the search for complementary clinical constructs, including progression independent of relapse activity [15].

### 2.2. The Artificial Boundary Between RRMS and SPMS

The transition from RRMS to SPMS represents one of the most consequential milestones in the disease course of multiple sclerosis. Clinically, SPMS is defined by a sustained accumulation of disability, independent of relapses, following an initial relapsing–remitting phase. Despite its conceptual clarity, the diagnosis of SPMS remains fundamentally retrospective, often established only after years of gradual deterioration have become unmistakable [3,16].

In real-world practice, the delay between the onset of progressive worsening and formal recognition of SPMS has been estimated to range from three to seven years. During this interval, patients are frequently still labeled as having RRMS, even as they experience steady functional decline. This diagnostic lag has significant clinical implications, as it may postpone therapeutic reassessment, limit access to treatments approved for progressive disease, and reduce opportunities for early intervention aimed at slowing progression [17,18].

The artificial nature of the RRMS–SPMS boundary becomes apparent when disease evolution is examined longitudinally rather than categorically. Disability accumulation does not typically begin abruptly at the moment SPMS is diagnosed; instead, it often emerges gradually, with periods of subtle worsening interspersed with relative stability. The eventual designation of SPMS reflects a threshold effect—a point at which progression becomes sufficiently obvious and sustained to satisfy clinical criteria—rather than a true biological transition [2,11,19].

This perspective challenges the notion that RRMS and SPMS represent distinct disease entities. Instead, it suggests that they may constitute different phases along a continuum of disease expression, governed by shifting balances between focal inflammatory activity, diffuse neurodegenerative processes, and compensatory mechanisms. Within this framework, the RRMS–SPMS distinction functions primarily as a descriptive label, capturing dominant clinical features at a given time point, rather than as a marker of discrete underlying biology [20,21].

The concept of progression independent of relapse activity further exposes the limitations of a rigid phenotypic boundary. PIRA demonstrates that progressive disability accumulation can occur well before the formal diagnosis of SPMS, undermining the assumption that RRMS is exclusively relapse-driven [22]. From this standpoint, SPMS may be better understood as the clinical culmination of accumulated, often unrecognized progression, rather than as the onset of a new disease phase [9,11,22].

Recognizing the artificiality of the RRMS–SPMS boundary does not negate the clinical utility of phenotypes. Rather, it underscores the need to reinterpret them within a dynamic, time-dependent model that acknowledges early progression and accommodates continuous disease evolution. This conceptual shift sets the stage for integrating PIRA into phenotype-based thinking and for redefining progression as a process that transcends traditional categorical distinctions [3,15,20].

## 3. PIRA: Redefining Progression

### 3.1. Definition and Emergence of PIRA

For decades, disability accumulation in multiple sclerosis was interpreted largely through the lens of inflammatory disease activity, with clinical relapses serving as the principal surrogate for disease progression. Seen through this lens, long-term worsening of neurological function was assumed to reflect either incomplete recovery from relapses or processes confined to later progressive stages of disease [10]. This relapse-centered model shaped both clinical reasoning and therapeutic development, reinforcing the notion that suppression of relapses equated to effective control of disease evolution [9].

Longitudinal observations increasingly challenged this view. Cohort studies and registry-based analyses showed that a substantial proportion of disability accumulation occurs in the absence of overt clinical relapses. Patients classified as stable RRMS, with relapse freedom and no new focal inflammatory activity on conventional MRI, were nonetheless found to experience sustained functional decline over time, suggesting that additional biological processes contribute to worsening [2,11,23].

Progression independent of relapse activity (PIRA) emerged to operationalize this phenomenon in a clinically meaningful way. PIRA refers to confirmed disability worsening occurring independently of clinical relapses, typically defined by sustained deterioration on disability measures over a predefined interval in the absence of temporally related relapses [15]. Rather than denying relapse-related disability accrual, PIRA isolates a component of progression not directly attributable to acute inflammatory events [2].

Conceptually, PIRA represents a shift from an event-based to a process-based understanding of disease progression. Whereas relapses are discrete and time-limited, PIRA captures a more continuous trajectory of worsening that may reflect underlying progressive pathology. This implies that progression may coexist with relapsing disease from early RRMS stages rather than emerging only after transition to SPMS [9,24].

The formalization of PIRA also reflects changes in the therapeutic landscape. As highly effective disease-modifying therapies reduced relapse rates and focal MRI activity, traditional inflammatory outcomes became less sensitive to ongoing disease evolution. In this context, PIRA provided a framework to detect disability accumulation despite apparent inflammatory control and is increasingly used in clinical trials and observational studies [7,25].

Crucially, PIRA is an operational construct rather than a single biological mechanism. It likely reflects heterogeneous processes contributing to neuroaxonal injury and functional decline, including compartmentalized inflammation, chronic microglial activation, mitochondrial dysfunction, and impaired remyelination [9,15,26,27].

The emergence of PIRA has also prompted a re-evaluation of traditional disease phenotypes. Detection of PIRA in patients with RRMS challenges the binary separation between relapsing and progressive disease courses and undermines the assumption that progression begins only at the transition to SPMS [11,15]. Instead, PIRA suggests that progressive pathology may be present long before it becomes clinically dominant, with phenotypic labels reflecting the relative balance between focal inflammatory activity and diffuse progressive processes at any given time [15,24,28].

Taken together, PIRA represents an important advance in MS research. By decoupling disability accumulation from relapses, it exposes limitations of phenotype-based classification and provides a clinical window into otherwise silent progression underlying long-term neurological decline [9,29].

### 3.2. Reversible vs. Persistent PIRA: Heterogeneity Within Relapse-Independent Progression

Although PIRA has been increasingly recognized as a major contributor to disability accumulation in multiple sclerosis, it is often implicitly treated as a uniform phenomenon. In practice, however, relapse-independent worsening is heterogeneous, both in its temporal dynamics and in its long-term clinical consequences [9,15]. Emerging longitudinal data suggest that not all PIRA events represent irreversible progression [27,30].

Several observational studies have shown that some patients meeting operational criteria for PIRA subsequently stabilize or partially improve. This suggests that, in a subset of cases, relapse-independent worsening may reflect transient dysfunction rather than permanent structural damage. Potential contributors include incomplete remyelination, fluctuating conduction block in chronically demyelinated axons, metabolic stress, and dynamic network reorganization [3,8,15].

In contrast, persistent PIRA is characterized by sustained disability accumulation beyond the initial confirmation window without meaningful recovery. This pattern is more consistent with irreversible neuroaxonal loss and progressive pathology, and is associated with worse long-term outcomes, including faster disability accrual and earlier transition to clinically defined SPMS [27,30].

This distinction may be interpreted through the concept of neurologic reserve. Transient PIRA may occur when progressive biological processes are partially compensated by reserve capacity and adaptive plasticity, such that worsening becomes clinically visible only when compensatory mechanisms are temporarily overwhelmed. Persistent PIRA, by contrast, may reflect exhaustion of compensatory capacity, allowing progressive pathology to become clinically dominant [2,31,32].

Such a framework also provides a bridge to the conventional transition from RRMS to SPMS. Rather than representing an abrupt biological shift, SPMS may reflect the stage at which persistent, uncompensated progression becomes sufficiently evident to meet clinical recognition thresholds [11,33].

Furthermore, most current operational definitions of PIRA do not distinguish between transient and persistent forms. Worsening is typically confirmed over relatively short intervals of three to six months, which may be insufficient to capture longer-term trajectories. As a result, biologically distinct patterns may be grouped within a single category [15,25].

Recognizing this heterogeneity has practical implications. In research, failure to distinguish transient from persistent PIRA may weaken associations with biomarkers, imaging measures, and long-term outcomes. In clinical practice, labeling all relapse-independent worsening as definitive progression may oversimplify prognosis and complicate therapeutic decision-making [34,35].

**Figure 1:**
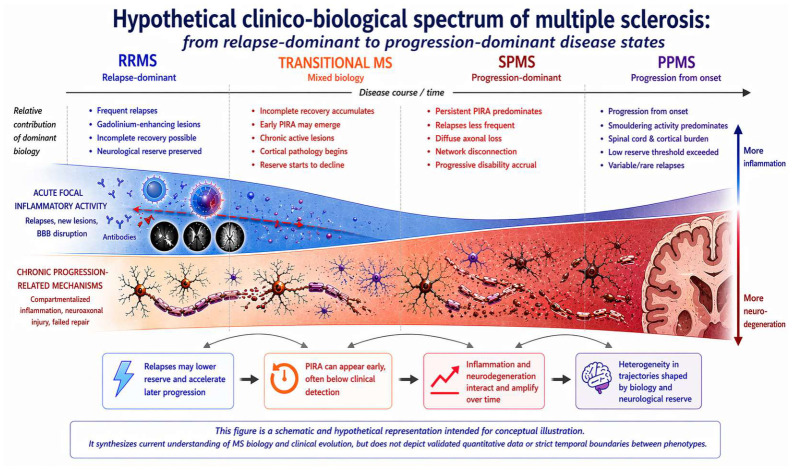
Clinical phenotypes as time-dependent expressions of shared disease biology in multiple sclerosis. This figure represents a conceptual schematic intended to illustrate hypothetical shifts in the relative contribution of inflammatory and progression-related mechanisms across the disease course. It is not based on quantitative modeling and does not imply specific rates, confidence intervals, or deterministic trajectories.

In summary, PIRA may be better viewed as a spectrum ranging from transient, potentially reversible worsening to persistent progression driven by irreversible pathology. This interpretation aligns with multiple sclerosis as a dynamic continuum in which clinical expression reflects the balance between tissue injury and compensatory reserve over time, as illustrated in Figure 1.

RRMS, SPMS, and PPMS are conceptualized as time-dependent clinical expressions of shared underlying disease mechanisms. Early disease is characterized by inflammation-dominated injury and predominantly transient relapse-independent worsening, whereas persistent progression independent of relapse activity (PIRA) becomes increasingly apparent with declining neurologic reserve. Transitions between phenotypes are illustrated as threshold phenomena rather than discrete biological shifts.

### 3.3. Operational Definitions and Sources of Heterogeneity in PIRA

Despite its conceptual utility, PIRA remains an operational construct whose implementation varies substantially across studies. Differences in outcome measures, confirmation intervals, relapse attribution rules, and approaches to subclinical inflammatory activity contribute to wide variation in reported PIRA rates and its estimated contribution to disability accumulation [36].

One major source of heterogeneity is the choice of disability metrics used to define worsening. Most studies rely on the Expanded Disability Status Scale, either alone or combined with functional tests such as the Timed 25-Foot Walk or 9-Hole Peg Test. These measures capture different neurological domains and vary in sensitivity to early or domain-specific decline [10,37]. Studies that depend primarily on EDSS are inherently biased toward detecting ambulatory worsening, whereas composite definitions incorporating upper limb or performance-based measures may identify a broader spectrum of relapse-independent deterioration [38].

A second important source of variability is the confirmation interval required to classify worsening as PIRA. Most studies use confirmation windows of three to six months, reflecting a compromise between feasibility and diagnostic certainty. Shorter intervals increase sensitivity but risk misclassifying transient fluctuations, whereas longer intervals improve specificity at the cost of delayed recognition [9,15,29].

Attribution of worsening to relapse-independent mechanisms introduces further complexity. Most definitions require the absence of a clinical relapse within predefined time windows before and after the worsening event, yet the optimal duration of these exclusion periods remains uncertain. Reliance on overt relapse definitions may also fail to capture subclinical inflammatory activity contributing to functional decline [29,39].

The handling of subclinical disease activity is particularly challenging. Conventional MRI detects focal inflammatory lesions but has limited sensitivity for diffuse or compartmentalized pathology. Consequently, worsening classified as PIRA may still occur in the presence of ongoing biological activity not captured by standard imaging [14,26,31].

Population characteristics further influence observed PIRA frequencies. Age, disease duration, treatment exposure, and baseline disability all modify the likelihood of detecting relapse-independent worsening and its consequences. Cohorts treated with highly effective disease-modifying therapies may show a higher relative proportion of PIRA because focal inflammatory activity has been suppressed, whereas untreated or historical cohorts may demonstrate a different balance between relapse-associated and relapse-independent worsening [12,23].

Altogether, these methodological variations highlight an important limitation of current PIRA frameworks: they treat relapse-independent worsening as a binary outcome despite substantial heterogeneity in underlying drivers and prognostic significance. Definitions relying on short confirmation intervals and limited outcome measures are poorly equipped to distinguish reversible deterioration from sustained progression driven by irreversible pathology [9,15,40].

These limitations do not invalidate the concept of PIRA, but emphasize the need for refinement and standardization. Future frameworks should incorporate multidimensional outcomes, longer-term trajectory assessment, and complementary biomarkers capable of capturing underlying biological activity. Such advances would improve comparability across studies, enhance clinical utility, and strengthen the role of PIRA as a bridge between clinical observation and progressive disease biology [25,34].

#### Practical Indicators Suggestive of PIRA

Given the heterogeneity of current definitions, no single clinical, imaging, or biomarker measure has been validated as diagnostic of progression independent of relapse activity (PIRA). In practice, PIRA is inferred from converging evidence of sustained worsening occurring outside the context of acute relapse activity and confirmed over time.

A pragmatic clinical framework may include: (1) objective disability worsening without a temporally related relapse; (2) confirmation of progression on repeat assessment after a predefined follow-up interval; and (3) supportive evidence from paraclinical or biomarker measures when available. Clinical worsening may be captured through longitudinal change in validated disability or performance measures, while progressive cognitive decline may represent an important manifestation, particularly in patients with limited change on conventional ambulatory scales [10,41,42].

Paraclinical investigations may provide complementary evidence. MRI markers such as accelerated brain or spinal cord atrophy, chronic active lesions, and slowly expanding lesions may support the presence of ongoing progression-related pathology [27,30,43]. Optical coherence tomography (OCT), particularly progressive retinal nerve fiber layer or ganglion cell-inner plexiform layer thinning, may offer an accessible marker of neuroaxonal loss [44,45].

Fluid biomarkers remain an evolving area of interest. Serum neurofilament light chain (NfL) may reflect ongoing neuroaxonal injury, while glial fibrillary acidic protein (GFAP) has been associated with astroglial activation and progressive disease features in some studies [46,47,48,49]. However, these markers currently remain adjunctive rather than definitive tools for identifying PIRA at the individual patient level.

Overall, PIRA should currently be regarded as a clinically inferred construct supported by multimodal evidence rather than a condition diagnosable by any single test. Further refinement and prospective validation will be required before formal diagnostic criteria can be established.

### 3.4. Biological Substrates Across the Multiple Sclerosis Spectrum

The clinical heterogeneity of multiple sclerosis is paralleled by substantial biological heterogeneity. RRMS, SPMS, and PPMS remain useful descriptive categories, yet increasing evidence suggests that their differences reflect shifting contributions of shared pathological processes over time [50]. The MS spectrum can therefore be viewed as a dynamic interaction between focal inflammation, compartmentalized tissue injury, repair capacity, and neurologic reserve.

#### 3.4.1. RRMS-Predominant Biology

Early relapsing disease is typically characterized by acute focal inflammatory activity driven by peripheral immune cell infiltration across a disrupted blood–brain barrier. This biology underlies new T2 lesions, gadolinium-enhancing lesions, and clinical relapses, which remain hallmark features of RRMS in many patients [51]. During this stage, remyelination, synaptic plasticity, and functional network reorganization may partially compensate tissue injury, allowing substantial recovery after relapses and relative preservation of neurological function [52].

However, inflammatory dominance does not imply biological uniformity. Even in early RRMS, diffuse axonal injury, cortical pathology, and low-grade compartmentalized inflammation may coexist with overt focal activity. These processes may remain clinically silent or only subtly expressed, particularly in individuals with preserved reserve capacity [31,53,54]. This helps explain why relapse suppression alone does not always prevent long-term disability accumulation.

#### 3.4.2. Transitional Biology

As the disease evolves, the balance between acute inflammation and progressive pathology may gradually shift. In many patients, this intermediate state is characterized by incomplete repair after inflammatory injury, cumulative lesion burden, and declining efficiency of compensatory mechanisms. Clinical recovery becomes less complete, while subtle relapse-independent worsening becomes more apparent [10,16].

Pathologically, chronic active lesions, persistent microglial activation, meningeal inflammation, and cortical demyelination assume increasing importance during this phase. These mechanisms promote continuous neuroaxonal injury beyond the boundaries of acute focal lesions and may contribute to early PIRA before formal recognition of SPMS [55,56]. The transition is therefore better understood as a gradual reweighting of dominant processes rather than an abrupt biological switch.

#### 3.4.3. Progressive-Dominant Biology (SPMS/PPMS)

In later or progressive-dominant stages, compartmentalized inflammation within the central nervous system becomes a central driver of tissue injury. Unlike acute peripheral inflammatory activity, these processes occur behind a relatively intact blood–brain barrier and are less visible to conventional inflammatory markers [51,53,57]. Chronic microglial activation, oxidative stress, mitochondrial dysfunction, impaired remyelination, and diffuse axonal degeneration contribute to sustained disability accumulation [58].

At this stage, network disconnection and loss of reserve capacity become increasingly clinically relevant. Functional compensation is reduced, so previously silent or partially compensated injury manifests as persistent progression [10,13,59].

#### 3.4.4. Mechanistic Determinants of Divergent Disease Courses

The clinical divergence between RRMS, SPMS, and PPMS is unlikely to arise from a single pathogenic switch. Rather, current evidence supports a model in which disease course reflects the relative timing, anatomical distribution, and intensity of several interacting mechanisms: peripheral immune activation, compartmentalized CNS inflammation, lesion topography, repair efficiency, neuroaxonal vulnerability, and reserve capacity. In this view, phenotypes are not discrete biological entities, but neither are they biologically identical; they represent differently weighted expressions of a shared pathogenic repertoire [59,60].

In RRMS, the dominant early signal is usually peripheral immune activation with episodic blood–brain barrier disruption. Autoreactive T cells, B cells, monocytes, and antigen-presenting cells enter the CNS through activated vascular and border-zone interfaces, producing focal inflammatory demyelination. Clinically, this biology is expressed as relapse: a temporally clustered inflammatory event occurring in a functionally eloquent CNS region [51,58]. Recovery is possible because inflammation may resolve, edema subsides, conduction block improves, and remyelination or network compensation can partially restore function. Thus, the relapsing-remitting pattern reflects not only inflammatory attacks, but also preserved CNS capacity for repair and compensation [52,61].

SPMS may be understood as the point at which this compensatory architecture progressively fails. Repeated inflammatory injury leaves behind chronically demyelinated axons, altered glial states, and increasingly inefficient remyelination. Over time, inflammation becomes less dependent on overt blood–brain barrier leakage and more compartmentalized behind a relatively intact barrier. Chronic active lesions, meningeal inflammation, cortical demyelination, diffuse normal-appearing white and gray matter injury, mitochondrial dysfunction, oxidative stress, and network disconnection increasingly dominate the clinical picture. In this setting, relapses may become less frequent, but disability continues to accumulate because structural and metabolic reserve has been progressively exhausted [53,55,60].

PPMS may reflect an early predominance of this progression-prone architecture rather than the late emergence of a distinct mechanism. Several factors may bias disease toward progressive expression from onset, including older age at manifestation, lower overt inflammatory burst activity, greater spinal cord or cortical involvement, reduced repair efficiency, lower baseline reserve, and stronger contribution of innate immune and glial mechanisms. Because spinal cord and cortical networks have limited capacity for functional compensation, relatively modest tissue injury in these regions may produce early sustained disability [62,63].

Recent genetic and systems-biology data further support this weighted-spectrum interpretation. Classical MS susceptibility loci predominantly implicate immune regulation, whereas emerging studies of disease severity suggest additional influences on CNS resilience, cortical pathology, disability worsening, and risk of secondary progression. These observations indicate that the biology determining whether MS begins may only partially overlap with the biology determining how it progresses [64,65].

Accordingly, RRMS, SPMS, and PPMS may be best conceptualized as biologically overlapping but differently weighted disease courses. RRMS is weighted toward episodic focal inflammatory activity and recoverable dysfunction; SPMS toward cumulative failure of repair and reserve after years of inflammatory and smouldering injury; and PPMS toward early expression of compartmentalized, glial, spinal cord, cortical, and reserve-limited pathology. This framework preserves the clinical reality of distinct disease courses while avoiding the misleading implication that they represent entirely separate diseases.

### 3.5. Interplay of Relapse-Associated Worsening and PIRA

Although relapse-associated worsening and progression independent of relapse activity (PIRA) are often discussed as separate contributors to disability accumulation, the underlying biology is unlikely to be strictly binary. Rather, these processes may overlap, interact, and reinforce one another across the disease course [10,66].

Relapses can produce lasting disability through incomplete recovery after acute focal inflammatory injury. However, even when overt recovery occurs, relapses may still contribute to cumulative tissue damage by reducing neurologic reserve and increasing vulnerability to subsequent decline [10,19,52]. In this way, the clinical impact of a relapse may extend beyond the immediate event.

Conversely, mechanisms typically associated with progression may also influence relapse outcomes. Chronic microglial activation, impaired remyelination, mitochondrial dysfunction, and pre-existing axonal injury may limit recovery capacity after new inflammatory lesions, making relapse-related deficits more likely to persist [52,57,61].

These observations suggest that relapse-associated worsening and PIRA should be viewed as partially distinct but biologically interconnected processes rather than mutually exclusive categories. Their relative contribution may vary between individuals and across disease stages, but both can converge on the shared pathway of neuroaxonal loss and functional decline [10,51].

Recognizing this interplay cautions against rigid attribution of worsening events to a single mechanism. Acute inflammatory activity, progressive pathology, and reserve capacity often interact simultaneously to shape long-term disability outcomes in multiple sclerosis.

## 4. Smouldering MS and SAW

### 4.1. Smouldering MS as a Biological Concept

The recognition of relapse-independent progression has prompted renewed interest in the biological processes that drive continuous neurological decline in multiple sclerosis. While PIRA provides a clinical framework to capture progression in the absence of relapses, it does not specify the underlying pathological mechanisms. In this context, the concept of smouldering multiple sclerosis has emerged as a unifying biological model to explain sustained disease activity beyond focal inflammatory events [67].

Smouldering MS refers to a state of chronic, low-grade inflammatory and neurodegenerative activity that persists within the central nervous system independently of acute relapses. Unlike classical inflammatory lesions characterized by blood–brain barrier breakdown and gadolinium enhancement, smouldering pathology is spatially compartmentalized and temporally sustained. It is thought to evolve behind a relatively intact blood–brain barrier, rendering it less accessible to both immune surveillance and conventional anti-inflammatory therapies [68,69].

One of the most characteristic pathological substrates of smouldering MS is the presence of chronic active (slowly expanding) lesions, often identified radiologically as paramagnetic rim lesions. These lesions exhibit ongoing microglial activation at their borders, accompanied by gradual expansion and progressive tissue damage over time. Unlike acute lesions, which may stabilize or partially remyelinate, chronic active lesions represent sites of continuous neuroaxonal injury and are increasingly associated with disability progression and long-term clinical outcomes [55,70].

Beyond focal lesion pathology, smouldering MS encompasses diffuse processes that affect normal-appearing white and gray matter. Widespread microglial activation, mitochondrial dysfunction, oxidative stress, and impaired energy metabolism contribute to a global environment of vulnerability within the central nervous system. These mechanisms promote gradual axonal loss and synaptic dysfunction, even in regions distant from overt lesions, thereby undermining neural network integrity [69,71].

Meningeal inflammation has also been implicated as a key driver of smouldering disease activity, particularly in progressive forms of MS. Ectopic lymphoid-like aggregates within the meninges are thought to sustain compartmentalized immune responses, releasing pro-inflammatory mediators that diffuse into adjacent cortical tissue. This process has been linked to subpial cortical demyelination and accelerated neurodegeneration, offering a potential explanation for the close association between cortical pathology and progressive disability [56,72].

It is worth emphasizing that smouldering MS is not restricted to clinically progressive phenotypes. Increasing evidence suggests that smouldering pathological processes are present from the early stages of the disease, including RRMS, where they may coexist with focal inflammatory activity. In such cases, acute relapses may dominate the clinical picture initially, masking the contribution of slower, progressive mechanisms. As disease duration increases and compensatory capacity declines, smouldering pathology becomes increasingly unmasked, contributing to relapse-independent disability accumulation [3,31,67].

The smouldering MS framework, therefore, provides a biological substrate for understanding PIRA. While PIRA captures the clinical expression of progression independent of relapses, smouldering MS describes the underlying pathological processes that drive this progression. The two concepts operate at different levels—clinical and biological, but are inherently linked. Smouldering pathology offers a mechanistic explanation for why disability may accumulate despite effective suppression of acute inflammatory activity.

Overall, the concept of smouldering MS represents a critical advance in reconciling clinical observations with neuropathological insights. It shifts the focus from discrete inflammatory events to continuous disease activity and highlights the need for models of MS that accommodate both focal and diffuse mechanisms of tissue injury. This biological perspective sets the stage for a more precise discussion of smouldering-associated worsening and its relationship to PIRA.

### 4.2. Smouldering-Associated Worsening (SAW)

To bridge biological concepts of smouldering disease activity with clinical observations of progression, the term smouldering-associated worsening (SAW) has been proposed. SAW refers to the gradual accumulation of neurological disability driven by chronic, compartmentalized pathological processes, rather than by acute inflammatory events. Unlike PIRA, which is defined operationally at the clinical level, SAW is intended to capture the biological substrate of slow, ongoing tissue injury [31,67,68].

SAW encompasses mechanisms such as chronic microglial activation, slowly expanding lesions, meningeal inflammation, and diffuse neuroaxonal damage affecting both white and gray matter. These processes evolve over extended timeframes and may remain clinically silent until compensatory mechanisms are exceeded. As a result, SAW provides a pathophysiological explanation for disability accumulation that appears disconnected from relapses and conventional MRI markers of acute inflammation [68,69,73].

Notably, SAW does not imply a uniform or linear trajectory. Its clinical expression is modulated by factors such as neurologic reserve, lesion distribution, and network vulnerability. Consequently, similar degrees of smouldering pathology may produce markedly different clinical outcomes across individuals. This variability mirrors the heterogeneity observed in relapse-independent progression and reinforces the need to distinguish biological activity from its clinical manifestations [67].

By conceptualizing progression as the cumulative effect of smouldering pathology, SAW reframes disability accumulation as an ongoing process rather than a discrete event. This standpoint aligns with emerging evidence that progressive mechanisms operate throughout the disease course, including during early RRMS, and are not restricted to clinically progressive phenotypes.

### 4.3. PIRA and SAW: Complementary, Not Competing Concepts

Although PIRA and SAW are sometimes used interchangeably, they operate at fundamentally different levels and should be regarded as complementary rather than competing constructs. PIRA is a clinical descriptor, defined by observed disability worsening in the absence of relapses, whereas SAW is a biological framework, describing the pathological processes that drive such worsening [14].

This distinction is critical for interpreting both clinical data and disease mechanisms. PIRA captures when progression becomes clinically detectable, but it does not specify why it occurs. SAW, by contrast, offers a mechanistic explanation but does not inherently define clinical thresholds or timing. In this sense, PIRA can be viewed as the clinical surface of an underlying smouldering disease process, which may remain subclinical for prolonged periods [9,14,15,31].

The imperfect overlap between PIRA and SAW helps explain observed heterogeneity in relapse-independent progression. Transient PIRA may occur in the context of smouldering pathology that is partially compensated by neurologic reserve, whereas persistent PIRA likely reflects sustained SAW exceeding compensatory capacity. Conversely, SAW may be present without immediate clinical expression, particularly in early disease stages or in individuals with high reserve [2,11,27].

Recognizing the complementary nature of PIRA and SAW has practical implications. Clinically, it cautions against equating the absence of PIRA with biological quiescence. From a research perspective, it underscores the limitations of relying solely on clinical outcomes to infer disease mechanisms. Integrating clinical constructs such as PIRA with biological markers of smouldering activity offers a more coherent framework for understanding progression across MS phenotypes. The complementary yet distinct nature of these constructs is summarized in Table 2.

## 5. Neurologic Reserve and the Continuum Model

### 5.1. Concept of Neurologic and Cognitive Reserve

The concept of neurologic reserve offers a critical explanatory framework for the marked interindividual variability observed in multiple sclerosis. Patients with comparable lesion burden and similar radiological findings often exhibit strikingly different clinical trajectories, suggesting that structural damage alone does not fully determine functional outcome. Neurologic reserve refers to the capacity of the central nervous system to compensate for injury through adaptive mechanisms, including network reorganization, synaptic plasticity, and recruitment of alternative neural pathways [41,74].

Closely related is the notion of cognitive reserve, which reflects the brain’s ability to maintain cognitive performance despite structural pathology. Factors such as premorbid intelligence, education, occupational complexity, and lifelong cognitive engagement are thought to enhance reserve, thereby buffering the clinical impact of neuroaxonal damage. In MS, higher reserve has been associated with delayed onset of cognitive impairment and reduced clinical expression of structural brain damage [75,76].

From this perspective, disability emerges not solely from the accumulation of pathology, but from the dynamic interaction between tissue injury and compensatory capacity. Smouldering pathological processes may therefore remain clinically silent for extended periods, particularly in individuals with high reserve. This helps explain why biologically active disease may not immediately translate into detectable progression on clinical scales.

### 5.2. Ageing, Exhaustion of Reserve, and Phenotype Shift

Clinical experience consistently shows that disability progression in multiple sclerosis relates more strongly to patient age than to disease duration. Relapse frequency typically declines with advancing age, whereas irreversible disability accumulation becomes more prominent. This observation challenges the traditional interpretation of a temporal transition from relapsing to progressive disease and instead suggests a change in host susceptibility to ongoing pathology [74,77].

Chronological age alone, however, does not adequately explain the variability of progression. Patients of similar age and lesion burden may follow markedly different trajectories, indicating that the relevant variable is biological ageing of the central nervous system rather than time elapsed since disease onset. Biological ageing reflects cumulative alterations in tissue resilience, including immunosenescence, microglial priming, mitochondrial dysfunction, reduced remyelination capacity, and vascular changes. These processes collectively lower the capacity of neural networks to compensate structural injury [78,79].

Within this framework, inflammatory demyelinating events and diffuse tissue damage may occur throughout the disease course, but their clinical expression depends on the available functional reserve. Early in the disease, compensatory mechanisms such as cortical reorganization, synaptic plasticity, and adaptive network recruitment maintain stable neurological function despite accumulating injury. With advancing biological ageing, these mechanisms become progressively insufficient. The same degree of tissue damage that was previously clinically silent begins to produce persistent deficits [52,80].

PIRA can therefore be interpreted as the clinical manifestation of a mismatch between ongoing pathological activity and declining compensatory capacity. Rather than representing the onset of a new pathological phase, it reflects the point at which reserve can no longer buffer injury. The clinical diagnosis of secondary progression emerges when this imbalance becomes sustained and functionally evident over time [10,81].

This model also explains the apparent distinction between relapsing and primary progressive disease. Instead of distinct biological entities, they may represent different starting positions along a shared continuum of tissue vulnerability. Individuals with reduced baseline reserve or accelerated biological ageing may manifest progressive disability earlier, whereas others maintain a relapsing phenotype for longer despite comparable underlying pathology [59,74].

Consequently, the transition between RRMS, SPMS, and PPMS may be better understood as a point of clinical decompensation driven by an ageing-related decline in CNS resilience rather than by the emergence of a qualitatively different disease mechanism. The phenotypes thus represent time-dependent clinical expressions of a continuous pathological process interacting with a gradually changing host substrate. Future integration of biological ageing markers with longitudinal PIRA assessment may help refine individualized progression thresholds [77,82].

## 6. Re-Interpreting RRMS, SPMS and PPMS Through PIRA

### 6.1. RRMS with Early PIRA: Subclinical Progression

The recognition of PIRA has fundamentally altered the understanding of disability accumulation in relapsing–remitting multiple sclerosis. Traditionally, RRMS has been conceptualized as a predominantly inflammatory disease course, with disability driven primarily by relapses and their incomplete recovery. Within this model, the absence of relapses has often been equated with disease stability. However, accumulating evidence indicates that relapse-independent progression may be present from early stages of RRMS, challenging the assumption that progression is confined to later disease phases [10,66].

Longitudinal cohort studies have demonstrated that a substantial proportion of disability accumulation in RRMS occurs independently of relapses. Intriguingly, PIRA events have been documented in patients with low relapse activity and even in those achieving apparent inflammatory control under disease-modifying therapy. These observations suggest that progressive disease biology may operate in parallel with focal inflammation, rather than emerging only after inflammatory activity subsides [10,66,83].

In early RRMS, relapse-independent worsening is frequently subtle and may escape detection by conventional clinical measures. Declines in upper limb function, cognitive efficiency, endurance, or complex motor performance may precede overt EDSS progression, particularly in patients with preserved ambulation. As a result, early PIRA often manifests as subclinical progression, detectable only through sensitive outcome measures or longitudinal assessment rather than through isolated clinical evaluations [84].

The presence of early PIRA has important implications for disease interpretation. RRMS patients without relapses should not be assumed to have biologically inactive disease, as relapse suppression does not preclude ongoing neuroaxonal injury. Instead, early PIRA may reflect the clinical expression of smouldering pathological processes that are partially compensated by neurologic reserve. In this setting, disability accumulation remains limited or fluctuating, delaying formal recognition of progression [66,70].

This framework also helps explain the variability in long-term outcomes among RRMS patients. Individuals with early and persistent PIRA are more likely to experience accelerated disability accumulation and earlier transition to SPMS, whereas those in whom relapse-independent worsening remains transient or minimal may maintain functional stability for prolonged periods. Thus, early PIRA may serve as a prognostic signal, identifying patients at higher risk of progressive disease evolution despite a relapsing clinical phenotype [10,81,85].

Conceptualizing RRMS as a disease course in which progressive mechanisms may already be active underscores the limitations of rigid phenotype-based thinking. Rather than representing a purely inflammatory stage, RRMS may be better understood as a phase in which the relative contribution of focal inflammation and smouldering pathology varies across individuals and over time. Within this spectrum, early PIRA provides a critical clinical window into the otherwise silent progression that shapes long-term disability trajectories [51,53].

### 6.2. SPMS as Accumulation of Persistent PIRA

Secondary progressive multiple sclerosis has traditionally been viewed as a distinct disease phase characterized by a qualitative shift from inflammatory to degenerative pathology. However, the emerging recognition of PIRA—particularly its persistent form—supports a more continuous interpretation of disease evolution. Rather than marking the onset of a new biological process, SPMS may represent the clinical culmination of sustained, relapse-independent worsening that has accumulated over time [10,81,82].

Within this framework, SPMS is best understood as the stage at which persistent PIRA becomes the dominant driver of disability. Unlike transient relapse-independent worsening, which may fluctuate or partially recover, persistent PIRA reflects ongoing neuroaxonal injury that exceeds compensatory capacity. As such, the defining feature of SPMS is not merely the presence of progression, but the loss of meaningful recovery and the consolidation of irreversible disability [82,86,87].

This perspective helps explain why the transition to SPMS is often gradual and difficult to pinpoint clinically. Persistent PIRA may be present for years before it becomes sufficiently evident to satisfy formal diagnostic criteria. During this period, patients may continue to be classified as having RRMS despite experiencing steady functional decline. The eventual diagnosis of SPMS thus reflects a threshold effect, where cumulative relapse-independent worsening becomes clinically unmistakable rather than biologically novel [17,81,87].

Importantly, this model reconciles heterogeneity in SPMS onset and progression rates. Patients with early, sustained PIRA are more likely to transition to SPMS at a younger age and with lower inflammatory activity, whereas those in whom PIRA remains intermittent or well-compensated may maintain a relapsing phenotype for prolonged periods. Differences in neurologic reserve, lesion distribution, and treatment exposure further modulate this trajectory, contributing to the wide interindividual variability observed in clinical practice [19,83,88,89].

Reframing SPMS as the accumulation of persistent PIRA also has implications for disease monitoring. It suggests that the biological processes underlying SPMS are already active during earlier disease stages and that waiting for formal SPMS diagnosis may delay recognition of meaningful progression. From this standpoint, SPMS should be viewed less as a categorical transition and more as a clinically recognizable endpoint along a continuum of relapse-independent progression [10,81,90].

By positioning SPMS within a spectrum model anchored in PIRA dynamics, this approach bridges classical phenotypic classification with contemporary insights into disease biology. It underscores the need to identify and characterize persistent PIRA earlier, before progression becomes entrenched and therapeutic opportunities narrow.

### 6.3. PPMS as Early Predominance of Smouldering Biology

Primary progressive multiple sclerosis has historically been regarded as a distinct disease entity, defined by insidious disability accumulation from onset in the absence of clinical relapses. Within the framework of PIRA and smouldering-associated pathology, PPMS may be better viewed as a disease course in which progressive biological mechanisms predominate from the earliest stages [53,86,91].

Compared with RRMS and SPMS, PPMS is characterized by lower levels of overt focal inflammatory activity and a relative paucity of clinical relapses. Nevertheless, neuropathological and imaging studies indicate that smouldering processes—such as chronic microglial activation, diffuse axonal injury, and slowly expanding lesions—are prominent in PPMS. These mechanisms drive continuous neuroaxonal loss and functional decline, resulting in a clinical course dominated by relapse-independent progression [92,93,94].

From this perspective, the defining feature of PPMS is not the absence of inflammation, but a different balance between inflammatory and progressive components. Focal inflammation may be less clinically apparent or less temporally clustered, while smouldering pathology exerts a sustained influence on disability accumulation. As a result, progression becomes clinically evident early, without an antecedent relapsing phase [53,91,93].

The PIRA framework provides a useful lens through which to interpret this pattern. In PPMS, relapse-independent worsening is effectively present from disease onset, with minimal contribution from relapse-associated disability. This contrasts with RRMS, where PIRA may be initially masked by relapses and compensatory reserve, and with SPMS, where persistent PIRA emerges gradually over time. Thus, PPMS can be conceptualized as a position along the MS spectrum where persistent PIRA is dominant early, rather than as a categorically distinct condition [2,9,85,91,95].

This reinterpretation helps reconcile shared pathological features across MS phenotypes. Many biological processes implicated in PPMS—such as mitochondrial dysfunction, impaired remyelination, and compartmentalized inflammation—are also present in RRMS and SPMS, differing primarily in timing and relative contribution. Phenotypic divergence therefore reflects differences in disease dynamics rather than fundamentally different mechanisms [20,53,57,96].

Understanding PPMS within a spectrum model anchored in PIRA and smouldering biology has important implications. It supports a unified view of MS pathogenesis, emphasizes common therapeutic targets across phenotypes, and challenges rigid distinctions that may obscure shared disease processes. This reinterpretation does not negate phenotypic distinctions but reframes them as dominant clinical expressions within a shared pathological continuum. In this context, PPMS represents an early and unmasked expression of progressive MS biology, rather than a separate disease pathway.

## 7. Diagnostic Implications in the McDonald 2024 Era

### 7.1. Toward a Unified Disease Framework

Recent updates to diagnostic frameworks for multiple sclerosis reflect a gradual shift away from rigid phenotype-based thinking toward a more unified view of disease biology. Although the McDonald criteria were not explicitly designed to redefine disease phenotypes, their evolving emphasis on dissemination in space and time, alongside growing recognition of progression independent of relapses, aligns conceptually with spectrum-based models of MS [1,97,98].

Within this context, the traditional separation between relapsing and progressive disease courses becomes less absolute. Progressive pathology is increasingly acknowledged as a component that may be present across phenotypes, even when overt progression is not yet clinically apparent. The recognition of PIRA reinforces this view, highlighting that disability accumulation can occur independently of relapses and may therefore escape detection within relapse-centric diagnostic paradigms [10,14,24,59].

A unified disease framework does not imply abandonment of phenotypic labels, but rather their reinterpretation. RRMS, SPMS, and PPMS remain useful descriptors of dominant clinical patterns; however, they should not be assumed to correspond to discrete biological states. Instead, diagnostic reasoning may benefit from explicitly considering progression-related processes alongside inflammatory activity at all stages of disease [3,20,97].

In practice, this approach shifts diagnostic focus from categorical classification toward longitudinal disease behavior. Rather than asking whether a patient has transitioned from RRMS to SPMS, clinicians may increasingly ask whether relapse-independent progression is present, whether it is persistent, and how it interacts with inflammatory activity and functional reserve. Such questions are more closely aligned with the underlying mechanisms that drive long-term disability [16,28,84,90,97].

### 7.2. Earlier Recognition of Progression and Its Limitations

The integration of PIRA into diagnostic thinking offers an opportunity for earlier recognition of clinically meaningful progression. By explicitly monitoring disability worsening independent of relapses, clinicians may identify progressive disease biology before it becomes entrenched and before formal criteria for SPMS are met. This has potential implications for both prognostication and therapeutic decision-making [29,99].

However, several limitations must be acknowledged. Current clinical tools lack sensitivity for detecting early or domain-specific progression, particularly in patients with preserved ambulation. Reliance on EDSS-based confirmation remains problematic, and short confirmation intervals may conflate transient functional fluctuations with true progression [100,101].

These constraints underscore that PIRA should not be interpreted as a definitive diagnostic marker, but rather as a clinical signal that warrants closer monitoring and comprehensive assessment. Integrating PIRA with complementary measures—such as performance-based tests, imaging markers of smouldering pathology, and patient-reported outcomes—may improve its diagnostic utility and reduce misclassification [14,34,100,102].

Ultimately, the diagnostic value of PIRA lies not in replacing existing criteria, but in augmenting clinical vigilance for progression across phenotypes. By encouraging systematic attention to relapse-independent worsening, PIRA-oriented assessment supports a more nuanced and proactive approach to disease monitoring within contemporary diagnostic frameworks.

## 8. Therapeutic Implications: From Phenotype-Based to Biology-Based Decisions

### 8.1. Limitations of Relapse-Centric Treatment Strategies

The therapeutic landscape of multiple sclerosis has expanded substantially over the past two decades, with the advent of highly effective disease-modifying therapies capable of robustly suppressing relapse activity and focal inflammatory lesions. This success has reinforced a relapse-centric treatment paradigm, in which therapeutic efficacy is predominantly evaluated through reductions in relapse rate and conventional MRI activity. While this approach has transformed outcomes for many patients, it has also exposed important conceptual and practical limitations [96,103].

Accumulating evidence indicates that control of relapses does not necessarily translate into control of long-term disability accumulation. Disability progression may continue in the absence of clinical relapses and overt inflammatory MRI activity, particularly through relapse-independent mechanisms captured by PIRA. In such cases, traditional markers of treatment success may coexist with ongoing neuroaxonal injury and functional decline [31,46,66].

This disconnect is especially relevant in RRMS patients who remain relapse-free under therapy but demonstrate sustained worsening over time. When relapse suppression is equated with disease stability, subtle progression may go unrecognized, delaying therapeutic reassessment. The lack of clear guidance for managing relapse-independent worsening outside of formally defined SPMS further reinforces therapeutic inertia, potentially narrowing the window for meaningful intervention [10,11,66].

These limitations do not diminish the importance of inflammatory control, which remains a cornerstone of MS treatment. Rather, they highlight the need to contextualize relapse suppression within a broader therapeutic framework that explicitly considers progression-related biology and its clinical expression.

### 8.2. PIRA-Informed Therapeutic Thinking

Incorporating PIRA into therapeutic decision-making requires a shift from rigid phenotype-based algorithms toward biology-informed clinical reasoning. Instead of focusing exclusively on whether a patient meets criteria for a specific phenotype, clinicians may benefit from assessing whether relapse-independent progression is present, whether it is persistent, and how it evolves longitudinally [15,85,97].

In patients with RRMS who exhibit early or sustained PIRA, continued reliance on therapies optimized primarily for relapse prevention may be insufficient to address the dominant drivers of disability. In this context, detection of relapse-independent worsening should prompt a structured re-evaluation of therapeutic goals, closer monitoring, and consideration of alternative strategies. Importantly, this does not imply that current therapies are ineffective, but rather that their impact on progressive mechanisms may be incomplete [100,104,105].

A PIRA-informed framework also broadens the definition of treatment failure. Traditionally, therapeutic failure has been defined by breakthrough relapses or new inflammatory MRI activity [106]. By contrast, failure to prevent ongoing disability accumulation, even in the absence of relapses, represents a clinically meaningful outcome that warrants attention. Aligning therapeutic assessment with long-term disability trajectories may therefore improve prognostication and patient-centered care [24,66,89,107].

At the same time, caution is required. Not all relapse-independent worsening reflects irreversible progression, and premature escalation based on isolated or transient changes may expose patients to unnecessary risk [25,100]. Distinguishing persistent from reversible PIRA remains essential, underscoring the importance of longitudinal assessment and multidimensional outcome measures in guiding therapeutic decisions [15,29].

### 8.3. Representative Therapeutic Mechanisms in the Context of PIRA

Highly effective anti-inflammatory therapies, including agents targeting B cell–mediated immunity such as anti-CD20 monoclonal antibodies (ocrelizumab, ofatumumab) and sphingosine-1-phosphate (S1P) receptor signaling (fingolimod, siponimod, ponesimod) have demonstrated profound efficacy in suppressing relapse activity and focal inflammatory lesions. Nevertheless, clinical observations and long-term data indicate that relapse-independent disability accumulation may persist under these treatments, highlighting a partial dissociation between inflammatory control and progressive disease biology [103,107,108,109,110].

This observation does not suggest therapeutic failure, but rather reflects the complexity of MS pathophysiology. While focal inflammation is efficiently targeted, smouldering and compartmentalized processes—such as chronic microglial activation and diffuse neuroaxonal injury—may remain relatively unaffected. As a result, PIRA can emerge or persist despite optimal control of relapses, particularly as neurologic reserve declines over time [70,73,93].

In response to these limitations, increasing attention has been directed toward therapeutic strategies that more closely align with progression-related mechanisms. Approaches targeting innate immune activation and compartmentalized inflammation within the central nervous system exemplify this shift, although clinical evidence remains emerging [96,111]. Bruton’s tyrosine kinase (BTK) inhibitors, such as tolebrutinib, are under investigation for their ability to modulate both CNS-resident microglia and B cell lineages, aiming to reduce chronic neuroinflammation and progressive tissue injury. These small molecules are designed to penetrate the blood–brain barrier and directly affect CNS-compartmentalized immune responses, which are key drivers of progression independent of relapses [112,113]. Within this evolving landscape, PIRA serves as a clinically relevant signal identifying gaps between current therapeutic capabilities and the biological processes driving long-term disability. Long-term disability trajectories, rather than relapse metrics alone, may represent a more appropriate benchmark for future therapeutic success. While this framework may help refine future therapeutic stratification and trial design, current treatment decisions remain guided by approved indications and established evidence rather than by PIRA status alone.

### 8.4. Toward Phenotype-Agnostic but Biology-Aware Treatment Strategies

The integration of PIRA into therapeutic reasoning supports a move toward phenotype-agnostic but biology-aware treatment strategies. In this model, therapeutic decisions are informed less by categorical labels such as RRMS or SPMS and more by the dominant disease processes present at a given time—namely focal inflammation, smouldering pathology, and the patient’s compensatory capacity [12].

Such an approach acknowledges that similar biological mechanisms operate across phenotypes, differing primarily in timing and relative contribution. Consequently, principles traditionally associated with progressive MS may be relevant earlier in the disease course for selected patients exhibiting relapse-independent worsening. This understanding also emphasizes the need for outcome measures that capture progression alongside inflammatory activity, both in clinical practice and in clinical trials [10,28,53,100].

Ultimately, recognizing the limitations of current therapies underscores a substantial unmet need in multiple sclerosis. While relapse suppression remains essential, addressing progression-related biology represents the next frontier of therapeutic development. Within this context, PIRA functions not only as a clinical descriptor of disability accumulation, but also as a guidepost for rethinking therapeutic strategies and evaluating future interventions [7,8].

## 9. Research Gaps and a Proposed Operational Framework

Despite substantial advances in understanding multiple sclerosis progression, several critical gaps remain. Foremost among these is the lack of a unified approach to identifying and characterizing relapse-independent progression early in the disease course. While PIRA has improved clinical recognition of progression beyond relapses, its current operationalization remains heterogeneous and insufficiently linked to underlying biology [14,114].

One major gap concerns the distinction between transient and persistent relapse-independent worsening. Existing definitions typically rely on short confirmation intervals and limited outcome measures, which may fail to capture long-term trajectories. As a result, biologically distinct processes—reversible functional deterioration and sustained neuroaxonal loss—are often conflated. Refining PIRA to incorporate trajectory-based assessment represents a priority for both research and clinical practice [9,15,29,88].

A second gap relates to the integration of biological markers capable of reflecting progression-related pathology. Clinical measures alone provide an incomplete picture of disease activity, particularly in early or compensated stages. Biomarkers reflecting neuroaxonal injury and smouldering pathology offer promise but are not yet routinely incorporated into progression assessment. Bridging clinical constructs such as PIRA with biological readouts remains an essential unmet need [35,48,115].

Third, current approaches inadequately capture patient-relevant dimensions of progression. Disability accumulation often manifests in domains insufficiently represented by traditional scales, including upper limb function, cognition, fatigue, and endurance. Patient-reported outcomes and performance-based measures may provide complementary insight into early progression but lack standardization and validation for this purpose [101,116,117].

In response to these gaps, we propose a minimal operational framework for early recognition of progression across MS phenotypes. This framework is not intended as a validated diagnostic algorithm, but as a pragmatic guide for research and longitudinal clinical assessment. The proposed framework rests on three core components: systematic evaluation of relapse-independent disability trajectories over extended timeframes with an explicit distinction between persistence and reversibility of worsening [118], integration of complementary biological markers indicative of neuroaxonal injury and smouldering pathology [31,47,49,70], and incorporation of multidimensional clinical and patient-reported measures sensitive to non-ambulatory domains of function [119,120,121]. Crucially, this framework is phenotype-agnostic. Rather than relying on categorical labels, it emphasizes the dynamic interaction between progressive pathology, inflammatory activity, and neurologic reserve. Applied longitudinally, it may facilitate earlier identification of patients at risk for sustained progression, inform therapeutic decision-making, and improve stratification in clinical trials targeting progression-related mechanisms [53].

By aligning clinical observation with biological insight, such an approach has the potential to refine the use of PIRA as a clinically meaningful construct and to advance a more coherent understanding of progression across the MS spectrum. This framework is conceptual and intended to support hypothesis generation and earlier recognition strategies rather than to serve as a validated clinical tool. Prospective studies are required before implementation in routine practice.

## 10. Conclusions

The traditional classification of multiple sclerosis into RRMS, SPMS, and PPMS has provided a valuable clinical framework for decades, facilitating diagnosis, communication, and therapeutic decision-making. However, accumulating evidence indicates that these phenotypes do not fully capture the biological and temporal complexity of disease evolution. In particular, the recognition of progression independent of relapse activity has exposed fundamental limitations of relapse-centric models and rigid categorical thinking.

This review proposes a spectrum-based interpretation of MS phenotypes, integrating PIRA, smouldering-associated worsening, and neurologic reserve into a unified conceptual framework. Within this model, progressive pathology is not confined to later disease stages or progressive phenotypes, but may operate from early RRMS onward, often remaining clinically silent until compensatory capacity is exceeded. Phenotypic labels are thus best understood as time-dependent expressions of a shared disease biology, shaped by the evolving balance between focal inflammation, smouldering pathology, and reserve.

A key contribution of this framework is the distinction between transient and persistent relapse-independent worsening. Recognizing heterogeneity within PIRA helps reconcile variability in clinical trajectories and offers a plausible bridge between early relapse-independent progression and the eventual emergence of SPMS. Importantly, this distinction also highlights limitations of current operational definitions and underscores the need for longitudinal, multidimensional assessment of progression.

Reframing MS through a spectrum lens has direct implications for clinical practice. It shifts attention from retrospective phenotype transitions toward proactive identification of progression-related signals, encourages integration of complementary clinical and biological measures, and supports more nuanced therapeutic reasoning. While current therapies remain primarily optimized for inflammatory control, PIRA provides a clinically meaningful marker of residual unmet need and a guidepost for future therapeutic development.

In conclusion, viewing MS as a dynamic spectrum rather than a collection of discrete phenotypes offers a more coherent and biologically grounded understanding of disease progression. Integrating PIRA with concepts of smouldering pathology and neurologic reserve may facilitate earlier recognition of clinically relevant progression, improve patient stratification, and align therapeutic strategies more closely with the mechanisms driving long-term disability. This perspective provides a conceptual foundation for future research aimed at refining progression assessment and targeting the processes that underlie irreversible neurological decline across the MS continuum.

## Figures and Tables

**Table 1:** Conventional clinical phenotypes of multiple sclerosis and their practical diagnostic or classification basis in the era of the 2024 McDonald criteria [1,2,3].

Phenotype	Practical Basis for Diagnosis or Classification	Most Relevant Current Modalities	Key Limitation/Caveat
**RRMS**	Relapsing course with clinical attacks followed by partial or complete remission; once MS is diagnosed, a relapsing onset course supports RRMS as the practical clinical phenotype	Clinical history and examination; brain/spinal MRI demonstrating typical dissemination in space; optic nerve assessment now usable as a fifth topographic site via orbital MRI, OCT, or VEP; CSF OCBs or kFLC index; CVS and PRL may increase specificity in uncertain cases	RRMS is a clinical course descriptor, not a separate McDonald diagnostic algorithm; silent progression may already be present despite relapse control
**SPMS**	Retrospective recognition of gradual, sustained disability accumulation after an initial relapsing-remitting course, not better explained by relapses alone	Longitudinal clinical follow-up; serial EDSS and multidomain functional assessment; supportive MRI evidence of atrophy, chronic active lesions, or slowly expanding lesions; optional OCT and fluid biomarkers as adjuncts	There are still no universally validated formal diagnostic criteria for SPMS equivalent to McDonald criteria; diagnosis remains delayed and partly judgment-based
**PPMS**	Progressive neurological worsening from onset over ≥12 months, plus supportive evidence of dissemination in space/typical MS pathology under the unified 2024 framework	Clinical evidence of progression over time; MRI with typical lesions; under the 2024 revisions, progressive-onset MS is diagnosed within the same unified framework, with the note that two or more spinal cord lesions can fulfill DIS even without brain lesions; CSF OCBs or kFLC index; optic nerve, CVS, and PRL may provide additional support in appropriate cases	Progressive onset can overlap biologically with relapsing-onset MS; phenotype describes dominant clinical expression rather than a categorically distinct biology

Abbreviations: RRMS, relapsing-remitting multiple sclerosis; SPMS, secondary progressive multiple sclerosis; PPMS, primary progressive multiple sclerosis; PIRA, progression independent of relapse activity; MRI, magnetic resonance imaging; DIS, dissemination in space; EDSS, Expanded Disability Status Scale; OCT, optical coherence tomography; OCBs, oligoclonal bands; kFLC, kappa free light chain; CVS, central vein sign; PRLs, paramagnetic rim lesions; NfL, neurofilament light chain; GFAP, glial fibrillary acidic protein.

**Table 2:** Comparative features of PIRA and smouldering-associated worsening (SAW) as clinical and biological constructs of progression in multiple sclerosis.

Feature	PIRA (Clinical Construct)	SAW (Biological Construct)	References
**Conceptual level**	Operational clinical phenomenon	Pathophysiological process	[66,67]
**Primary focus**	Accumulation of measurable disability	Chronic compartmentalized CNS pathology	[66,67,73]
**Core definition**	Confirmed disability worsening independent of relapses	Ongoing tissue injury driven by smouldering inflammatory–neurodegenerative mechanisms	[36,66,67]
**Biological substrate**	Not directly specified	Microglial activation, chronic active lesions, diffuse axonal loss, meningeal inflammation	[56,68,69,70,71]
**Mode of detection**	Clinical scales and functional testing	Advanced MRI markers and neuropathology	[37,56,70]
**Typical temporal scale**	Months (confirmation intervals)	Years (cumulative damage)	[36,39,67]
**Relation to neurologic reserve**	Modulated by reserve and compensation	Largely independent of compensatory capacity	[41,74]
**Clinical implication**	Indicates progression has become clinically detectable	Explains why progression occurs biologically	[66,67]
**Reversibility**	May be transient or persistent	Intrinsically progressive	[15,17,18,67,73]
**Role in disease course**	Defines transition toward clinically progressive phenotype	Operates throughout disease continuum	[20,66,67,74]
**Key limitations**	Definition heterogeneity and dependence on clinical thresholds	Limited direct clinical observability in routine practice	[36,39,67]

## Data Availability

No new data were created or analyzed in this study.
